# Nutrition literacy is associated with income and place of residence but not with diet behavior and food security in the Palestinian society

**DOI:** 10.1186/s40795-021-00479-3

**Published:** 2021-11-18

**Authors:** Nihal Natour, Mariam AL-Tell, Osama Ikhdour

**Affiliations:** Department of Public Health, School of Medicine, An-Najah University, Nablus, Palestine

**Keywords:** Diet, Nutrition literacy, Obesity, Food security

## Abstract

**Introduction:**

Palestinian society is going through health transition that is associated with increase in chronic diseases due to poor dietary habits so adequate integration of nutrition information is important.

**Aims:**

The aim of this study is to find the association between nutrition literacy and diet behavior among a group of Palestinian participants.

**Methods:**

A sample of 101 Palestinian participants were recruited to participate in the study. An online survey was used to collect study data. Newest Vital Sign quiz was used to collect information on nutrition literacy and Short Format of the Diet Health and Knowledge Survey (SFDHKS) was used to collect information on diet behavior and USDA food security questionnaire was used to collect data on food security. Data was analyzed utilizing SPSS 21.

**Results:**

This study included 101 participants, mean age 22.7 y ± 8.7 y, mainly females (females were 83.2% and males were 16.8%). 5.7% of the study participants were obese, 13.8% overweight and 10.3% were underweight. The prevalence of adequate nutrition literacy was 29%. There was minimal association between diet behavior and nutrition literacy, food security and BMI categories, but significant association with income and living in city relative to village (*p* < 0.05). Only 11 participants had some form of food insecurity.

**Conclusion:**

There is low prevalence of adequate nutrition literacy. Nutrition literacy depends on social and economic aspects but further research is need to understand its relationship to diet behavior.

**Supplementary Information:**

The online version contains supplementary material available at 10.1186/s40795-021-00479-3.

## Introduction

Diet is a major risk factor for chronic diseases including type 2 diabetes mellitus, cardiovascular diseases and cancer. Chronic diseases rates have increased globally [1]. Poor dietary habits account for 16% of all-cause mortality in USA adults according to data from National Health and Nutrition Examination Survey (NHANES 1999–2010) and healthy eating index of 2015 [2]. Diet related problems including obesity account for increased health cost and lower quality of life [3, 4]. Dietary habits correlate with socioeconomic factors in different sections of the society, data from NHANES indicates that healthy eating index was 4 times higher for adults with high versus low education and 2 times higher for food secure versus food insecure [5].

Efforts to improve dietary habits in Palestine could be accomplished by enhancing nutrition knowledge. Food literacy, nutrition literacy and health literacy are concepts that reflect general knowledge about health and nutrition. Food literacy can be defined as the ability to understand, use, analyze and communicate nutrition information [6], whereas nutrition literacy means the ability of a person to seek, understand and apply basic nutrition information [7]. In one study among 386 American adults higher nutrition literacy significantly correlated with adherence to prudent diet (*P* < 0·001, *β* = 0·36), Mediterranean diet ((*P* = 0·02, *β* = 0·12) and was inversely related to adherence to westernized diet (*P* = 0·003, *β* = − 0·13) even after adjustment for covariates including age, BMI and gender [8]. On the other hand, poorer health literacy as measured by NVS was associated with increase in BMI ((*r* = − 0.12, *p* = .027), increase in age ((*r* = − 0.26, *p* < .001) and higher NVS measured literacy was more common in higher income and education [9]. Improving individuals’ access to information and their ability to use them is considered effective involvement of adults in managing their health [10]. Improving nutrition literacy could lead to better healthful diet decisions and can be achieved by many tools such as food labels and printed brochures and material to deliver nutrition information [11]. Literacy itself which is defined as “an individual’s ability to read, write, and speak in English and compute and solve problems at levels of proficiency necessary to function on the job and in society, to achieve one’s goals, and to develop one’s knowledge and potential.” Is important to use and apply distributed nutrition information [12].

The Palestinian society is in health transition associated with open food market with different countries [13]. In a recent study by a group of Palestinian researchers the prevalence of being overweight or obese is 65.3% and metabolic syndrome is 33% [14]. with current adoption of western lifestyle near crowded urban centers changes to generation dietary habits is prevalent in many countries including Palestine [15]. Having adequate nutrition knowledge could hinder the shift in obesity and chronic diseases epidemic in Palestine. The aim of this paper: 1) To study the level of nutrition literacy among a group of Palestinians using NVS 2) To study the relationship between dietary habits and nutrition literacy. 3) To study the differences in dietary habits according to BMI categories and food security categories.

## Methods

A cross-sectional design was used to evaluate nutrition literacy, food security and its association with dietary habits. Palestinians older than 18 y were recruited through an electronic data collection tool which was distributed through different social media methods that included facebook and professional, social and student facebook groups, in addition to the university website. The population consisted of all Palestinians living in the West Bank, Gaza, and in Israel. A convenient sampling method was adopted. sample size of *n* = 101 adults participated in this study. The data collection tool was adopted based on a Literature review [16, 17]. Information on age, weight, height,diet, use of food label, items of food label used,gender, education, income were obtained. The Newest Vital Sign (NVS) of 5 items was used to assess nutrition literacy [18]. NVS assessed participants to understand and calculate basic nutrition information using two nutrition fact labels obtained from Palestinian common products [18]. Each correct answer was given one point and a total score ranged from 0 to 5, and categorized as following 0–1 this indicated lack of nutrition literacy, 2–3 indicated possibility of limited nutrition literacy and 4–5 indicated adequate nutrition literacy. NVS was validated before [18]. A translated to Arabic version was used in our study which was reviewed by expertsThe food labels that were presented to participants were 1) a food label of commonly purchased biscuits that had information (in arabic and English) pertaining to serving size, total calories, percent daily value and grams per serving of total fat, saturated fat, cholesterol, transfat, carbohydrate including total sugars, added sugars, dietary fibers and proteins. 2) a food label from bagel salty snack that shows a list of ingredients in arabic in that food. Both pictures are attached as supplementary material.

Arabic translated version of dietary habits was used and consisted of nineteen questions from the Short Format of the Diet Health and Knowledge Survey(SFDHKS) [19] was used to measure food label use and diet behavior the translated tool was reviewed by experts. A short form of USDA food security scale was used and we used questions AD1 and AD1a after converting the answers to likert scale numbers to classify food security into food secure and insecure [20]..

### Statistics

The ordinal data for the answers of SFDHKS was converted into Likert scale numbers. Proportions of various sociodemographic, nutrition literacy, obesity and food security were calculated. Normality of continuous variables was calculated. Non-parametric test was used to compare SFDHKS, BMI and age between food secure and insecure and medians and range were reported. One way ANOVA was used to compare food behavior variables across food literacy groups and BMI categories. Chi-square was used to study association between categorical variables.

## Results

**Fig. 1:**
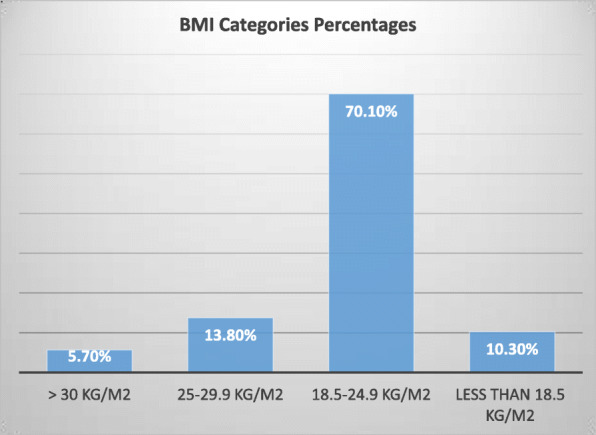
Percentages of Various BMI Categories. Chi-Square = 10.9, p-value = 0.027

This study included 101 participants, mean age 22.7 y ± 8.7 y. Relatively there was low prevalence of obesity, but higher prevalence of overweight and underweight participants (Fig. 1). NVS consisted of 5 questions; the first question asked the study group about the total calories in the whole package and for this question 66% had correct answers,2) the second question was if you were to take 60 g of carbohydrate how much of the product you will consume and for this 35% answered correctly 3) the third question what is the percent of total needed calories does one serving of this product provides based on 2000 kcal need, and for this only 35% answered this correctly. 4) The participants were asked if they would consume the product if they have allergy for soy and barely and for this 95% answered correctly and fifth question was if you have allergy why will you stop eating this product and for this 55% answered correctly.  Table 1 describes study main variables. The study group were mostly females, the prevalence of adequate literacy was slightly more than quarter of the study participants. Most of the study participants reported a household income of 3000–5000 shikel and having or doing bachelors degree. Only 11 participants reported some form of food insecurity. Also, mean of total nutrition literacy score was compared across study variables’ categories; Females, group with higher income, food label user and group who consumed low calorie products and looked on health benefits on food label had higher food literacy score (Table 1). Table 2 describes comparison of dietary habits between food literacy groups. Participants with adequate literacy reported lower use of high fat cheese, fried chicken and higher removal of chicken skin. Other variables were not significantly different between nutrition literacy. Table 3 show comparison of study variables between food secure and insecure adults, food secure were more likely to use mayonnaise and cheese as addition to their food and less likely to remove the skin of the chicken. Table 4 describe differences of study diet variables between BMI categories. Use of low fat milk was least common among adults with normal BMI. Underweight participants did not care to consume low fat and low calorie products.

**Table 1:** Distribution of participants’ percentage according to their demographic data

Variable	N (%)	Mean ± SD
Newest Vital Sign		
High Likelihood of Limited Health Literacy	19/103 (18.4%)	0.8 ± 0.4^a^
Possible Limited Health Literacy	54/103 (52..4%)	2.5 ± 0.5^b^
Adequate Health Literacy	30/103 (29.1%)	4.4 ± 0.5^c^
**Gender**		
Male	17/101 (16.8%)	2.3 ± 0.95^a^
Female	84/101 (83.2.%)	3.0 ± 1.1^b^
**Income**		
Less than 3000 Shikel	22/99 (22.2%)	2.2 ± 1.1^a^
3000–6000 Shikel	44/99 (44.4%)	3.2 ± 1.3^b^
More than 6000 Shikel	33/99 (33.3%)	2.9 ± 1.2^ab^
**Place**		
Refugee Camp	3/100 (3%)	2.5 ± 0.7
City	55/100 (55%)	2.7 ± 1.2
Village	42/100 (42%)	3.1 ± 1.4
**Education**		
High School or less	7/101 (7%)	3.3 ± 1.3
College	12/101 (11.9%)	2.5 ± 1.2
Bachelor	78/101 (77.2%)	2.9 ± 1.3
Postgraduate education	4/101 (4%)	2.3 ± 1.9
**Label USE**		
Use of food Label		
May be	24/101 (24%)	2.9 ± 1.1^ab^
No	33/101 (33%)	2.3 ± 1.1^a^
Yes	44/101 (43%)	3.3 ± 1.2^b^
Looking at food Label		
May be	16/98 (16.3%)42/98	2.9 ± 1.1^ab^
No	(42.9%)	2.4 ± 1.3^a^
Yes	40/98 (40.8%)	3.2 ± 1.3^b^
Look at health claim on food label		
May be	13/99 (13.1%)	2.9 ± 1.4
No	56/99 (56.6%)	2.8 ± 1.2
Yes	30/99 (30.3%)	2.7 ± 1.4
Look at calories on the food label		
May be	25/101 (24.8%)	2.7 ± 1.1^ab^
No	41/101 (40.6%)	2.5 ± 1.4^a^
Yes	35/101 (34.7%)	3.2 ± 1.3^b^
Look at serving size on food label		
May be	38/101 (37.6%)	2.4 ± 1.0
No	31/101 (30.7%)	2.1 ± 1.2
Yes	32/101 (31.7%)	2.4 ± 1.0
Look at health benefit on Label		
May be	35/101 (34.7%)	2.8 ± 1.1^ab^
No	39/101 (38.6%)	2.6 ± 1.4^a^
Yes	27/101 (26.7%)	3.4 ± 1.2^b^
**Food Security**		
Food Secure	89%	2.9 ± 1.3
Modest insecurity	8%	2.8 ± 1.4
Severe insecurity	3. %	2.5 ± 0.7

This table presents subcategories percentages and also it present NVS total score

mean ± SD for each categories, differences between means was calaculated using

ANOVA. Means with different letters are statistically significant

**Table 2:** Differences between the Food Literacy Groups in relation to the dietary variables by ANOVA/F test

Variable	Limited Literacy (N= 19)	Possible Limited Literacy (N=54)	Adequate Literacy (N=30)	F, *p*-value
Age (y)	23.0 ± 4.0	22.2 ± 7.7	22.8 ± 9.6	1.16, 0.32
BMI (Kg/m^2^)	22.7 ± 4.0	22.6 ± 3.7	22.1 ± 4.3	0.16, 0.85
Consumption of low-fat/low-calorie foods	1.86 ± 0.64	2.20 ± 0.89	2.30 ± 0.94	1.24, 0.30
Eat lower fat lunch meats	1.26 ± 1.1	2.1 ± 1.3	1.90 ± 1.2	2.56, 0.08
Drink skim or 1% milk	1.5 ± 1.3	1.6 ± 1.2	1.4 ± 1.4	0.04, 0.96
Low fat cheese	1.0 ± 1.13	1.69 ± 1.1	1.31 ± 1.39	2.26,.11
Use low calorie dressing	0.80 ± 0.7	1.54 ± 1.22	1.37 ± 1.24	2.36, 0.10
Eat fried Potatoes	2.8 ± 1.0	2.40 ± 1.2	2.60 ± 0.77	0.92, 0.40
Frying veggi	1.4 ± 0.7	1.5 ± 1.1	1.6 ± 1.1	0.09, 0.9
Adding cheese and mayonise	1.7 ± 0.8	1.6 ± 0.8	1.7 ± 0.8	0.8, 0.46
Eat butter, bread, cake	1.6 ± 1.3	1.8 ± 1.0	0.9 ± 0.9	2.9,0.07
Avoid extra fat	2.0 ± 1.3	2.5 ± 1.2	2.50 ± 0.90	1.27, 0.29
Fried chicken	2.47 ± 0.83	2.18 ± 1.1	1.36 ± 0.82	8.3, 0.000
Remove skin from chicken	2.47 ± 1.46	3.14 ± 1.23	3.21 ± 1.08	2.06, 0.13

Data expressed as means±SD. Differences were calculated using ANOVA

**Table 3:** Differences between Food Security Groups in relation to Study Variables using non-parametric test

Variable	Food secure *N* = 84	Food Insecure *N* = 11	Nonparametric, p-value
Age (y)	19(32)	20 (29)	0.18
BMI (Kg/m^2^)	21.6 (16.9)	22.7 (13.5)	0.56
Consumption of low-fat/low-calorie foods	2(4)	2(4)	0.52
Eat lower fat lunch meats	2(4)	1(4)	0.22
Drink skim or 1% milk	2 (5)	1(5)	0.65
Low fat cheese	1 (4)	1(4)	0.77
Frozen Yogurt	1 (5)	2(5)	0.11
Use low calorie dressing	1 (4)	1(4)	0.77
Eat fried Potatoes	3 (4)	2(4)	0.08
Frying veggi	2 (5)	2(5)	0.99
Adding cheese and mayonise	2(5)	3(5)	0.03
Eat butter, bread, cake	1 (5)	2(5)	0.24
Avoid extra fat	2 (4)	2(4)	0.1
Eat Fried chicken	2 (4)	2(4)	0.32
Remove skin	4 (4)	2(4)	0.08

Data is expressed as median (range). Differences between means were

Calculated using non-parametric test

**Table 4:** Differences between BMI categories in relation to Diet Behavior Variables by ANOVA test

Variable	Underweight (N=11)	Normal (N= 66)	Overweight and Obese (N=21)	F, *p*-value
Age (y)	19.0 ± 1.2	21.0 ± 6.2	30.5 ± 13.4	12.3, *p* < 0.0001
BMI (Kg/m^2^)	17.6 ± 0.7	21.3 ± 1.82	28.8 ± 2.30	171.4, p < 0.0001
Low calorie fat	1.64 ± 1.21	2.24 ± 0.84	2.24 ± 0.70	2.4, 0.09
Low fat meat	1.55 ± 1.13	1.89 ± 1.27	2.0 ± 1.11	0.50, 0.61
Low fat milk	2.46 ± 1.97	1.52 ± 1.37	2.65 ± 1.53	5.50, 0.006
Low fat cheese	1.18 ± 1.08	1.55 ± 1.34	1.38 ± 0.80	0.52, 0.6
Frozen Yogurt	1.56 ± 1.33	1.17 ± 1.54	1.35 ± 0.93	0.37, 0.70
Low calorie seasoning	1.18 ± 1.17	1.39 ± 1.23	1.38 ± 1.11	0.15,.86
Fried Potatoes	2.0 ± 1.34	2.59 ± 1.01	2.42 ± 1.07	2.39, 0.097
Frying veggi	2.1 ± 1.66	2.14 ± 1.90	1.43 ± 1.12	1.35, 0.27
Adding cheese and mayonise	2.20 ± 2.0	2.06 ± 1.70	2.10 ± 1.37	0.03, 0.97
Butter, bread, cake	1.46 ± 1.51	1.45 ± 1.46	1.48 ± 1.40	0.003, 0.99
Avoid fat	2.10 ± 1.22	2.60 ± 1.14	2.10 ± 0.89	2.26, 0.11
Fried chicken	2.36 ± 1.12	2.03 ± 1.10	1.67 ± 0.86	1.73, 0.18
Remove skin	3.36 ± 1.12	3.19 ± 1.18	2.57 ± 1.36	2.37, 0.099

Data is presented as mean ± SD, differences were calculated using ANOVA

Nutrition literacy was not different according BMI, food label, food security and gender categories. In multiple regression model (Table 5), being from a village significantly reduced total nutrition literacy score, whereas food security was strongly but not significantly related to nutrition literacy score.

**Table 5:** Multiple Regression Model of the Association between Total literacy score and Study Variables (*n* = 89)

Variable	B ± SE	t-value	p-value
Age	−0.02 ± 0.02	−1.0	0.30
Female versus male	−1.03 ± 0.94	−1.1	0.25
Income			
3000 shikel or less	−1.8 ± 1.5	− 1.2	0.23
3000–6000 shikel	−0.23 ± 1.5	−0.16	0.88
More than 6000 shikel	REF		
Place			
Village versus city	−2.5 ± 0.95	−2.5	0.035
Refugee camp versus city	−0.50 ± 0.9	− 0.55	0.58
Food security score	−0.15 ± 0.09	−1.8	0.079

B is beta coefficient of regression model and SE is standard error. Multiple regression model using total NVS score is dependent outcome in relation to gender, income, total food security score and place of residence,

## Discussion

This study involved participants from the Palestinian society from different age groups through online recruitment, however females and younger adults were more willing to participate in the study. In our study 29% of the study group had adequate nutrition literacy which is slightly lower than what was reported in other groups [17]. Our study group were low to middle income group, the group with higher income had better literacy compared to other groups. Studies and public campaigns to improve nutrition knowledge among the general Palestinian society and skills to read food labels and perform simple nutrition calculations are limited. We have performed a study on food label use among Palestinian group and found that although the use of food labels among Palestinian society is very common, they only seeked information on crude values of calories, sugar and fat without looking into important information such as sodium content and types of harmful lipids in food products [23]. Nutrition education in Palestinian society is imperative and should be supported with appropriate funding that direct resources on important tools such as raising awareness to nutrition comprehension skills among the various sectors of the society.

Similar to what others found, majority of study participants did not answer the calculation questions correctly. The first three question required document literacy and numeracy literacy skills but the other two did not require that. This is in accordance with what was found for a group of Americans which could indicate that numeracy represents a challenge for many [20]. Dietitian and nutritionists should perform more efforts in trying to simplify nutrition guidance which requires some numeracy skills by providing the client with more simplified information.

Income and whether the participant is from village or city were significantly related to nutrition literacy. Participants with higher income and from cities had higher nutrition literacy than participants from villages and lower income. Since most of our study participants were females this could reflectpatriarchy [24] than socio-demographic variables. Income gap and geographical locations of Palestinian villages could not justify discrepancies in terms of access to healthcare Many Palestinian villages are close to cities centers, and despite the fact Palestinian cities have higher concentration of nutrition care services, reaching out to these centers is feasible with the strong transportation system in the West Bank [25].

Most of our study participants did not use food label, or used information on them on calories, health benefits of the product. Less than half of our study group did not use low fat or low calorie diet or even low fat milk products, despite the fact that 50% reported using low fat meat. The low use of food label could be related to the fact that participants do not understand them, whereas the low purchase of low calorie or low fat milk products could be related to the fact they are expensive and this study group were mostly from low to middle income category [22, 26].

This study indicated low association between health literacy and food behavior. People with adequate literacy were more likely to eat reduced fat meat and to avoid eating fried chicken. One possible conclusion from this finding is that even people with adequate health literacy are unable to understand nutrition messages sent by nutrition practitioners in the society. In a previous study [27] under review, we found that participants from health field students were able to understand how to use MyPlate American application. However, they reported low efficiency in applying what they learn from this website on their practical life. This indicates that health care providers need to simplify nutrition by providing practical workshops with graphs and cooking skills to the participants who wish to change their diet style.

Income was a significant predictor of nutrition literacy as well as living place which could reflect the importance of socioeconomic status on nutrition literacy. Similar to this study, a study among Iranian adolescents showed that mother education, higher socioeconomic status were associated with higher nutrition knowledge scores [28]. This was further supported by other studies [29, 30]. On the other hand, similar to our study in multiple regression models there was no significant association between BMI and nutrition literacy score [28]. Studies on the association between BMI and nutrition literacy are inconsistent [30, 31] and may differ according to age and gender. Our study was more representative of young adults and females.

This study indicates that food insecurity was not an issue in the studied group of mostly young Palestinians Participants who had access to internet participated in the study so it is expected that food security was uncommon. Previous studies on food security in Palestinian society indicated that prevalence of severe food insecurity 24.6%. Food insecurity was related to poverty, unemployment, low education and having more than seven members in the family [32]. We did not find any common poor dietary habit among the 11 participants who reported some form of food insecurity, neither had we found differences in BMI category or food literacy.

We studied the relationship between BMI category and food habits and food literacy. Obesity and being underweight were not related to food literacy, whereas obese adults were more likely to practice healthier habits and underweight group were less likely to practice healthy habits. In a study among university female students obese, overweight and normal weight females did not have significant difference is dietary habits or sleeping duration [33]. Although obese youth may avoid some unhealthy food items, they may have unhealthy practices such as skipping breakfast [34].

### Study limitation

This study was planned and data was collected during corona lockdown. Due to movement restriction we decided to have convenient sample from those who responded to survey online through many websites including university website for students and social media. The response rate was lower than expected, one justification for that could be related to the fact this survey required some calculations and was long, so many individuals were not willing to participate. This could be a bias. Also, we did not collect data on diet intake using any tool such as food frequency questionnaire which could have provided a better view of food habits of study group. Another limitation of this study is its cross sectional design.

## Conclusion

In summary in a group of Palestinian adults mostly in their twenties and females, we found low prevalence of nutrition literacy (29%) and use of food label (43%). Many of the study group had unhealthy dietary habits but very few were food insecure. Nutrition literacy was related to diet behavior especially on the willingness of people with higher food literacy to use low fat and low calorie food products and they are also more frequent user of food label. Food insecurity group only had few unhealthy habits, whereas unhealthy dietary habits were common among underweight Palestinians.

## Supplementary Information

**Additional file 1.** .

## Data Availability

The datasets generated and/or analyzed during the current study are not publicly available due [being kept confidential for future work] but are available from the corresponding author on reasonable request.

## Abbreviations

USDA: United States Department of Agriculture
BMI: Body Mass Index
SFDHKS: short form of diet health and knowledge survey
NHANES: National Health and Nutrition Examination Survey
NVS: Newest Vital Sign
