# Supplementary figures and images for: Nutrition literacy is associated with income and place of residence but not with diet behavior and food security in the Palestinian society
Source: BMC Nutr. 2021 Nov 18;7:78. doi: 10.1186/s40795-021-00479-3 (PMC8600769; doi:10.1186/s40795-021-00479-3)

Supplementary File

Supplementary Material

1. Food Label 1


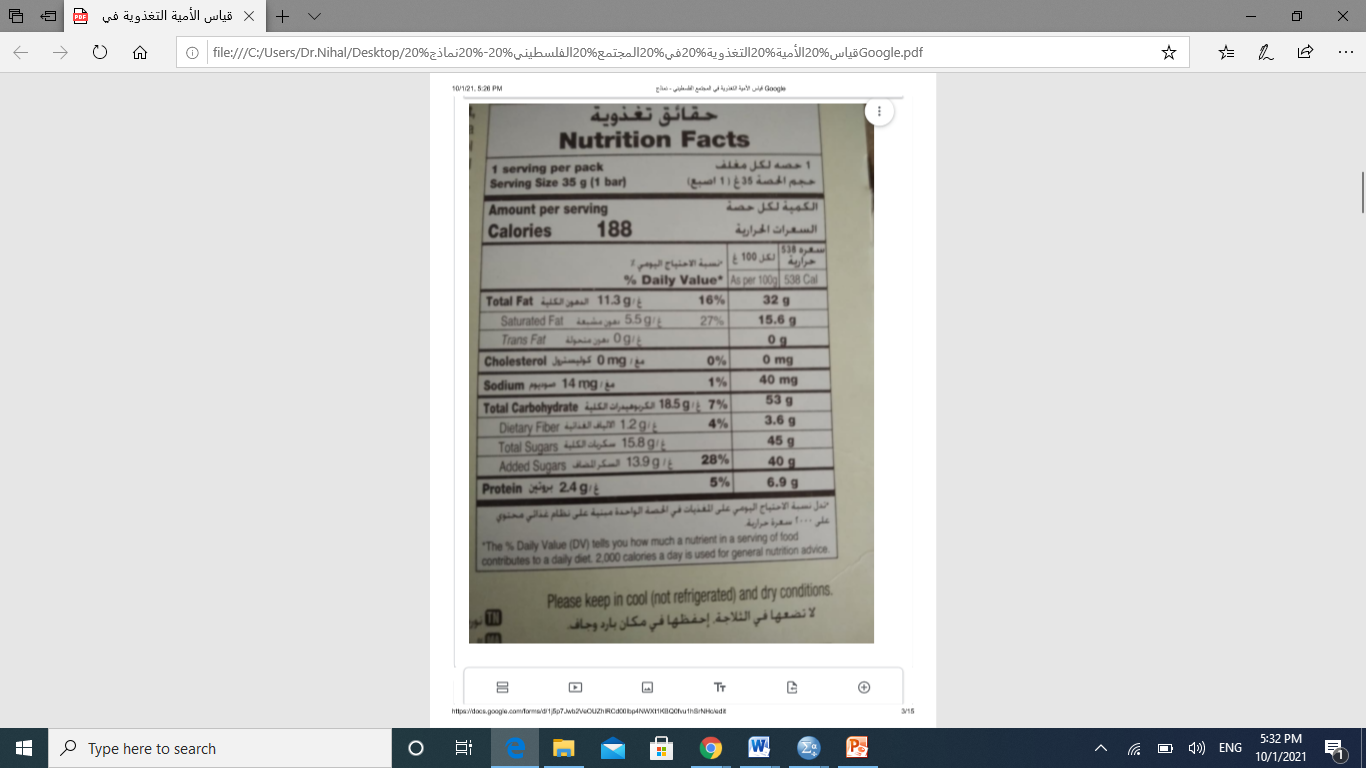


1. Food Label 2


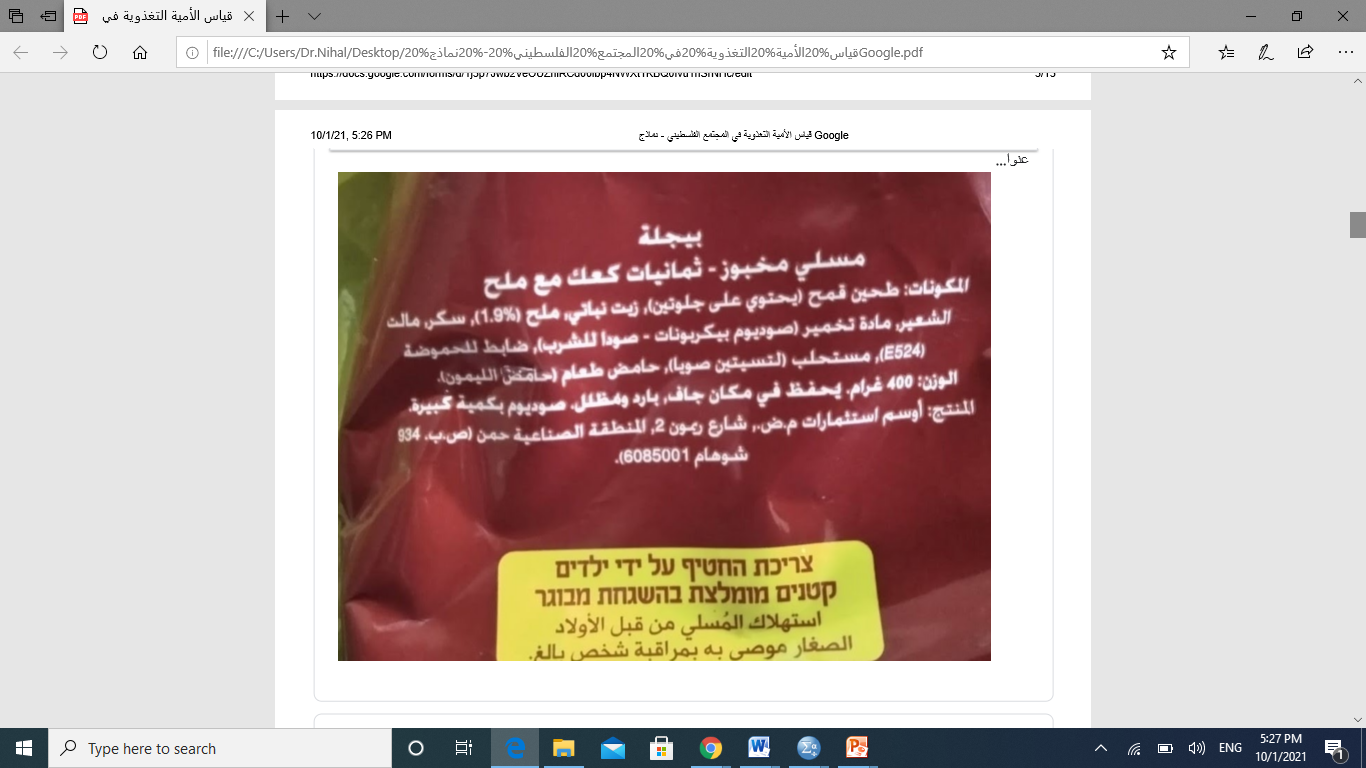

Supplement: Supplementary file 1 — Additional file 1. . [file 40795_2021_479_MOESM1_ESM.docx]
